# Commentary: Viewing photos and reading nouns of natural graspable objects similarly modulate motor responses

**DOI:** 10.3389/fnhum.2015.00337

**Published:** 2015-06-09

**Authors:** Stergios Makris

**Affiliations:** Department of Psychology, Edge Hill UniversityOrmskirk, UK

**Keywords:** objects, nouns, affordance, motor cortex, visual perception

Marino et al. (2014) in their paper (*Frontiers in Human Neuroscience*) have tried to investigate how the semantic processing of graspable objects involves an activation of the motor cortex in line with the affordance hypothesis originally proposed by Gibson (1979). They devised a go/non-go behavioral task, during which they presented photos or nouns of natural graspable and non-graspable objects, while for some of the trials the stimuli viewed were scrambled images of the same objects or pseudowords. Participants viewed the stimuli for a period of 150 ms, after which they had to respond to a go or non-go signal (whether the stimuli were real or not) as part of a semantic task. They found that subjects' responses were slower when they were viewing the photos or reading the nouns of graspable objects, as compared to non-graspable ones. The authors explained that this delay in motor responses following the images or nouns of graspable objects is a proof of the motor cortex involvement in the semantic processing of objects that afford a motoric action. Even though these findings are in line with some previous reports about an early activation and involvement of the motor system in language and semantic processes (Pulvermueller et al., 2001, 2005), in this commentary we argue that the stimulus onset asynchrony (SOA) of 150 ms is too early for an affordance effect to occur and thus, we will try to provide a different account of their results and leave some room for further insight on the topic.

There is mounting research evidence suggesting that the simple viewing of objects with action significance can stimulate the motor cortex into generating appropriate motor plans, even in cases that there is no action intention (Tucker and Ellis, 1998; Ellis and Tucker, 2000; Makris et al., 2011, 2013). This is the theory of affordances as originally described by Gibson (1979). Within the affordance literature a key aspect for investigation has been the temporal evolution of the affordance effect. Ellis and Tucker (2000) in a series of behavioral investigations have suggested that the affordance effect is slow and gradually develops 500 ms after the stimulus onset. On the other hand, in previous research with TMS we have proved by means of measures of corticospinal excitability (motor evoked potentials) that the affordance effect is present at 300 ms and rapidly dissipates 500 ms after the stimulus onset (Makris et al., 2011, 2013). Most importantly, in the aforementioned studies we investigated the generation of affordances 150 ms after the subjects were presented with graspable objects, but we did not find any evidence of involvement of the motor cortex as a result of that. In that sense, the results of Marino et al. (2014) are in contrast with previously reported findings.

Furthermore, Cisek (2006, 2007) has provided a compelling explanation of this delay in the formation of the affordance effect, known as the “affordance competition hypothesis.” According to this, in response to attended objects with action significance, multiple competing motor plans are generated across different regions of the motor cortex and through mutual inhibitory connections, a single motor winning act prevails. With this in mind, it is possible that graspable objects suddenly appearing on screen can automatically grab exogenous attention (Yantis and Jonides, 1984) and then for a rapid period after stimulus onset (~100–150 ms) attention is subsequently withdrawn from the objects in display, leading to a rebalance of the affordance-driven motor plans (see also Makris et al., 2011). This is particularly interesting, as it could provide an alternative explanation for the observed difference in response latencies between graspable and non-graspable objects in the Marino et al. (2014) study. Indeed, it could be that 150 ms after the presentation of the stimuli, exogenous-like attention was withdrawn from the graspable only objects and not the non-graspable ones. This way, participants would have to re-direct their attention to the graspable objects in order to resolve the semantic task and thus, this process would have some cost in the timing of their responses. Hence, the reported results may not reflect the involvement of the motor system in the semantic processing of graspable stimuli *per se*, but instead an effect of purely attentional processes. Nevertheless, this is only an alternative proposition to the current findings by Marino et al. (2014) and even so we cannot entirely rule out a relationship between attentional and motor processes (i.e., *premotor theory of attention*, Rizzolatti et al., 1994).

Overall, it is apparent that the affordance effect remains a compelling topic within cognitive psychology and neuroscience, as it is the need to better understand the underlying visual, attention and motor processes. Theories of a direct or indirect route between visual perception, semantic processing and motor planning may appear contradicting, but in our opinion it could be that they are all providing a valuable insight in the better understanding of human cognition and perception. Hence, it is important for future research to validate or expand upon these insights.

## Conflict of interest statement

The author declares that the research was conducted in the absence of any commercial or financial relationships that could be construed as a potential conflict of interest.
